# A Rare Case of Prosthetic Mitral Valve Endocarditis With Atrial and Ventricular Lead Infections

**DOI:** 10.7759/cureus.79713

**Published:** 2025-02-26

**Authors:** Mohamed Elhadi, Abdullah Motam, Aemen Khalid, Ravish Katira

**Affiliations:** 1 Gastroenterology, East Lancashire University Hospitals NHS Trust, Blackburn, GBR; 2 Gastroenterology, Royal Blackburn Hospital, Blackburn, GBR; 3 Nephrology, Royal Preston Hospital, Preston, GBR; 4 Cardiology, Mersey and West Lancashire Teaching Hospitals NHS Trust, Prescot, GBR

**Keywords:** acute hemorrhagic stroke, device-associated endocarditis, intravenous drug use (ivdu), prosthetic valve infective endocarditis, staphylcoccus aureus

## Abstract

This is the case of a patient in their 30s who is known to have a prosthetic mitral valve replacement and a cardiac pacemaker that presented to the hyper-acute stroke unit with collapse, left-sided dense weakness, back pain, dyspnea, and hypoxia. Investigations showed bilateral areas of intracerebral haemorrhage. Transthoracic echocardiogram (TTE) showed prosthetic mitral valve, atrial and ventricular lead vegetations with severe valvular incompetence due to the valve being markedly thickened with mobile oscillating masses seen on atrial and ventricular sides. The patient was not a candidate for surgical management given a history of continuous intravenous drug use (IVDU) as well as significant risks posed by haemorrhagic stroke and poor previous post-operative compliance. While the patient initially showed improvement with medical management, including appropriate antibiotics, persistent staphylococcus aureus bacteremia remained. Despite ongoing treatment efforts, the patient experienced clinical deterioration and succumbed to their illness from multi-organ failure. This case highlights the challenges in managing infective endocarditis involving prosthetic valves and cardiac devices, particularly in the setting of contraindications to surgical intervention and significant comorbidities. It highlights the need for a multidisciplinary approach to balance the risks of surgical versus medical management in complex cases, as well as the importance of early recognition and tailored therapeutic strategies.

## Introduction

Endocarditis is a life-threatening condition characterized by the inflammation of the endocardium, the inner lining of the heart, and is often associated with infection. It predominantly involves the heart valves, either native or prosthetic, and can also extend to other intracardiac devices. Infective endocarditis (IE) can lead to a range of serious complications, affecting multiple organ systems. Early recognition and management are crucial to mitigate these risks. Despite medical advancement, IE remains a highly lethal disease with an estimated number of worldwide deaths to be 66,320 in 2019 [1]. Complications may include cardiac, neurological, and pulmonary manifestations [2]. The primary mechanism relating IE to neurologic complications is the embolic spread of infected material from the affected area to the brain. Neurological complications following endocarditis significantly contribute to increased mortality and long-term morbidity in patients with IE [3]. The incidence of neurological complications in IE patients ranges from 20% to 55%, with ischemic stroke being the most common manifestation [4]. These neurological events are associated with a poor prognosis, including increased mortality and morbidity from disabling sequelae [4]. One retrospective multi-centre study reported haemorrhagic strokes were 7% [5], with the incidence of intracerebral haemorrhage (ICH) in IE varying between 7% and 27% [6]. Given the significant impact of neurological complications on outcomes, early detection, and management are crucial in patients with IE.

Prosthetic valve endocarditis and cardiac device-related IE represent unique clinical challenges due to their complex presentations, diagnostic difficulties, and significant morbidity and mortality. Prosthetic valve endocarditis constitutes 10-30% of all cases of IE and arises in individuals with implanted prosthetic heart valves [7]. *Staphylococcus aureus* has become the leading causative microorganism in prosthetic valve endocarditis [7]. The European Society of Cardiology recommends transthoracic echocardiography and transesophageal echocardiography for the evaluation of cardiac implantable electronic device (CIED) IE [8]. Additionally, 18F-fluorodeoxyglucose positron-emission tomography/computed tomography (18-FDG PET/CT) demonstrates high sensitivity and specificity, particularly in cases of suspected CIED IE [9]. Cerebral imaging is crucial to assess potential neurological complications of IE. Magnetic resonance imaging (MRI) with and without gadolinium contrast is the preferred modality. If MRI is not feasible, CT with and without contrast is an appropriate alternative [8].

This case highlights the serious nature of embolic phenomena as an initial presentation of IE, particularly in patients with persistent bacteremia, permanent pacemakers, and prosthetic valves. It highlights the increased mortality risk when surgical intervention is not feasible. Additionally, it emphasizes the challenges associated with embolic complications and the poor outcomes linked to non-compliance to postoperative instructions. The diagnostic limitations arising from an MRI-incompatible pacemaker further illustrate the difficulty in managing this case. 

## Case presentation

A patient in their mid-30s was transferred from a regional hospital to the Hyper Acute Stroke Unit (HASU) following a sudden collapse associated with reduced consciousness and a Glasgow Coma Scale score of 8. Initial observations on admission were notable for hypotension (blood pressure - 97/53 mmHg), tachycardia (heart rate - 123 beats/min), respiratory rate of (16 breaths/min), oxygen saturation of (94% on 5 L/min) of nasal cannula oxygen with no signs of impending respiratory failure with adequate oxygenation on the arterial blood gas, and a normal temperature 36.3°C. Clinical examination revealed left-sided dense weakness, mild dysarthria, reduced consciousness and systolic murmur alongside complaints of back pain. Relevant medical history was notable for intravenous drug use, permanent pacemaker and bioprosthetic mitral valve replacement for prior endocarditis. Table 1 shows increased inflammatory markers, low platelet, deranged electrolytes, liver function tests and kidney profile. The most concerning was thrombocytopenia in the context of ICH as it is associated with increased bleeding.

**Table 1:** Blood tests on admission showing abnormalities

Laboratory test	Admission tests	Reference range
White cell count	11.1	4.0 – 10.9 × 10^9/L
Platelets	52	150 – 450 × 10^9/L
C-Reactive protein	300	0 – 5 mg/L
Alanine transaminase	76	0-40 IU/L
Urea	9.3	2.5–7.8 mmol/L
Adjusted calcium	1.85	2.2–2.6 mmol/L
Phosphate	0.73	0.8–1.5 mmol/L

**Figure 1:**
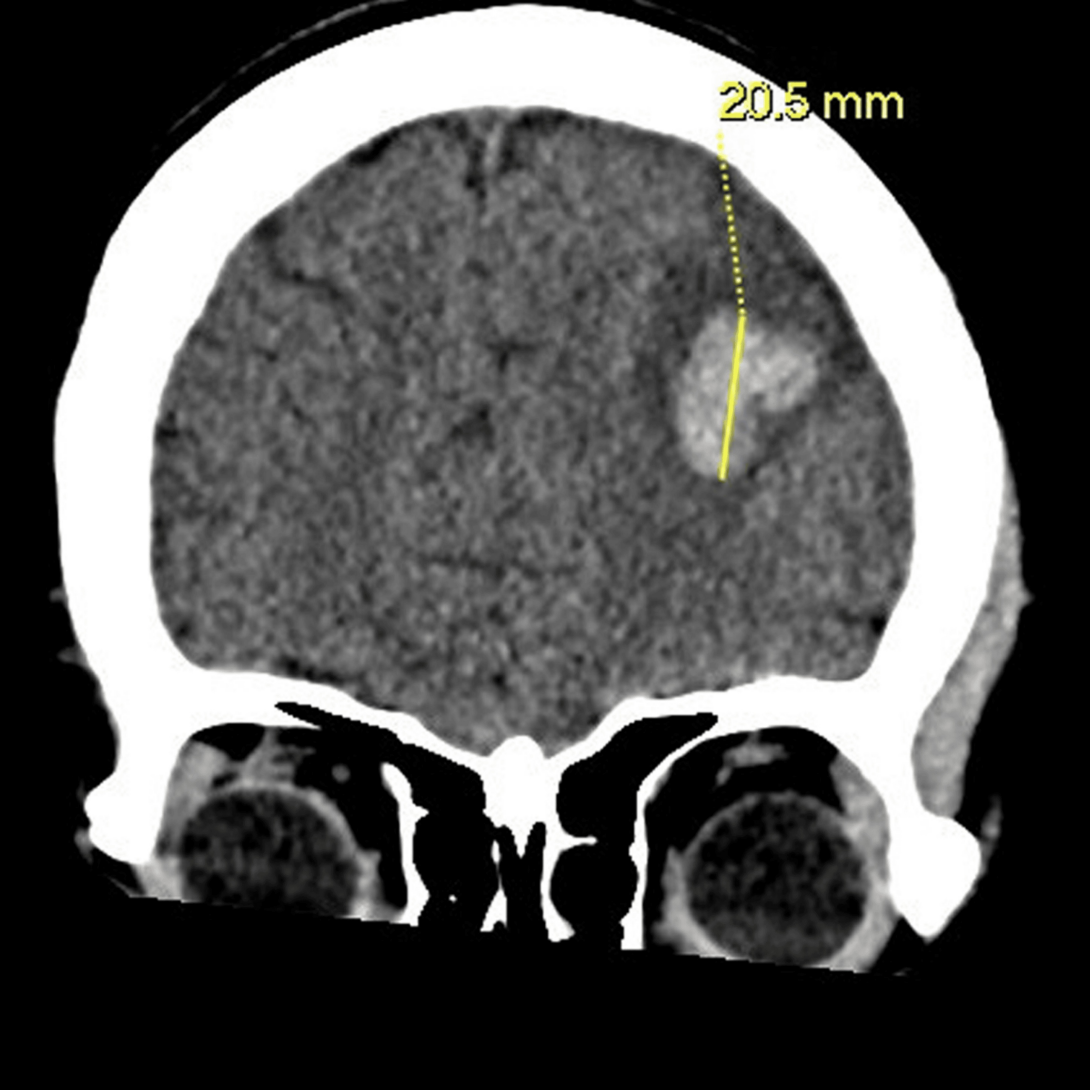
Coronal CT head imaging showing the area of brain haemorrhage

**Figure 2:**
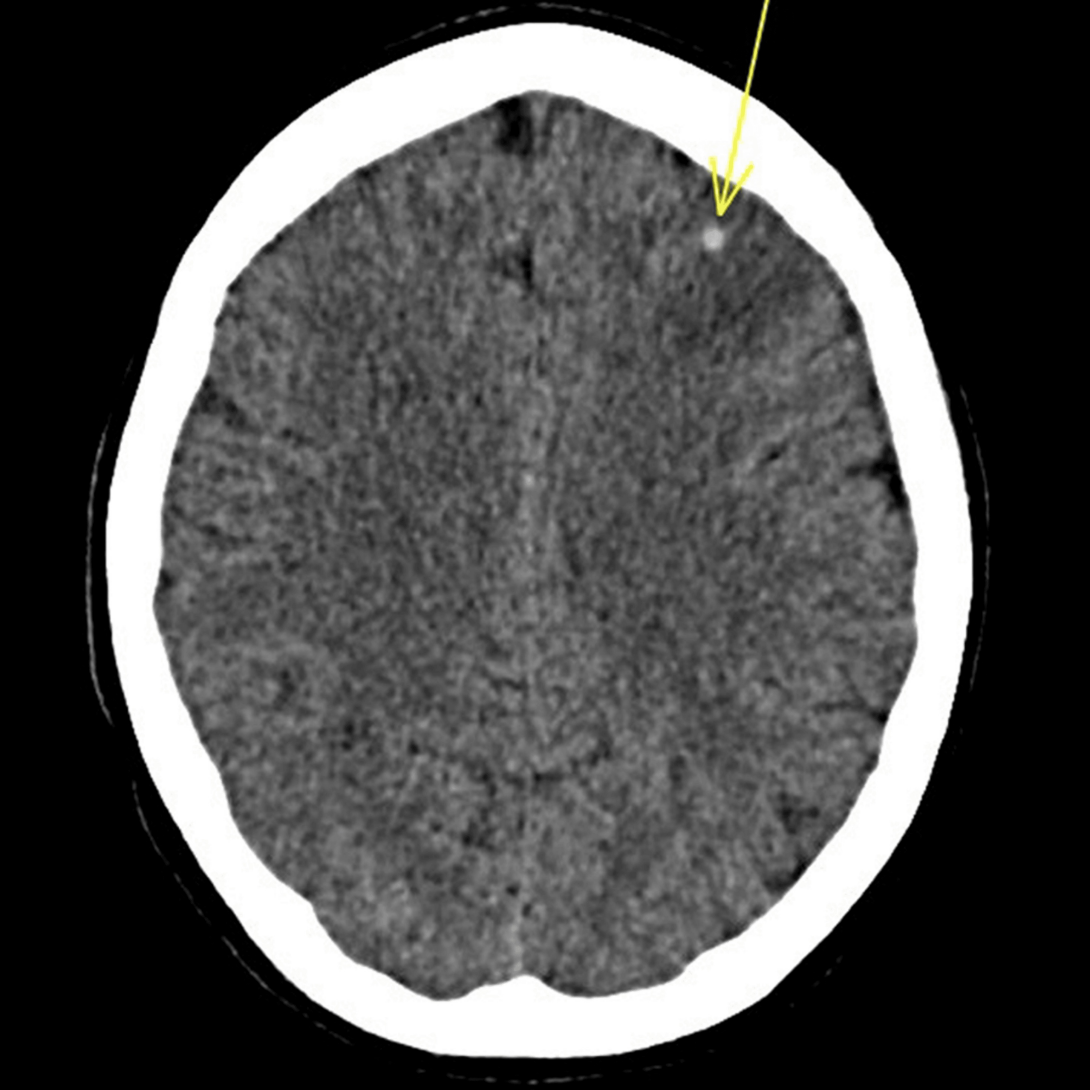
Axial CT head imaging showing a small area of brain micro-haemorrhage, highlighted by the yellow arrow

**Figure 3:**
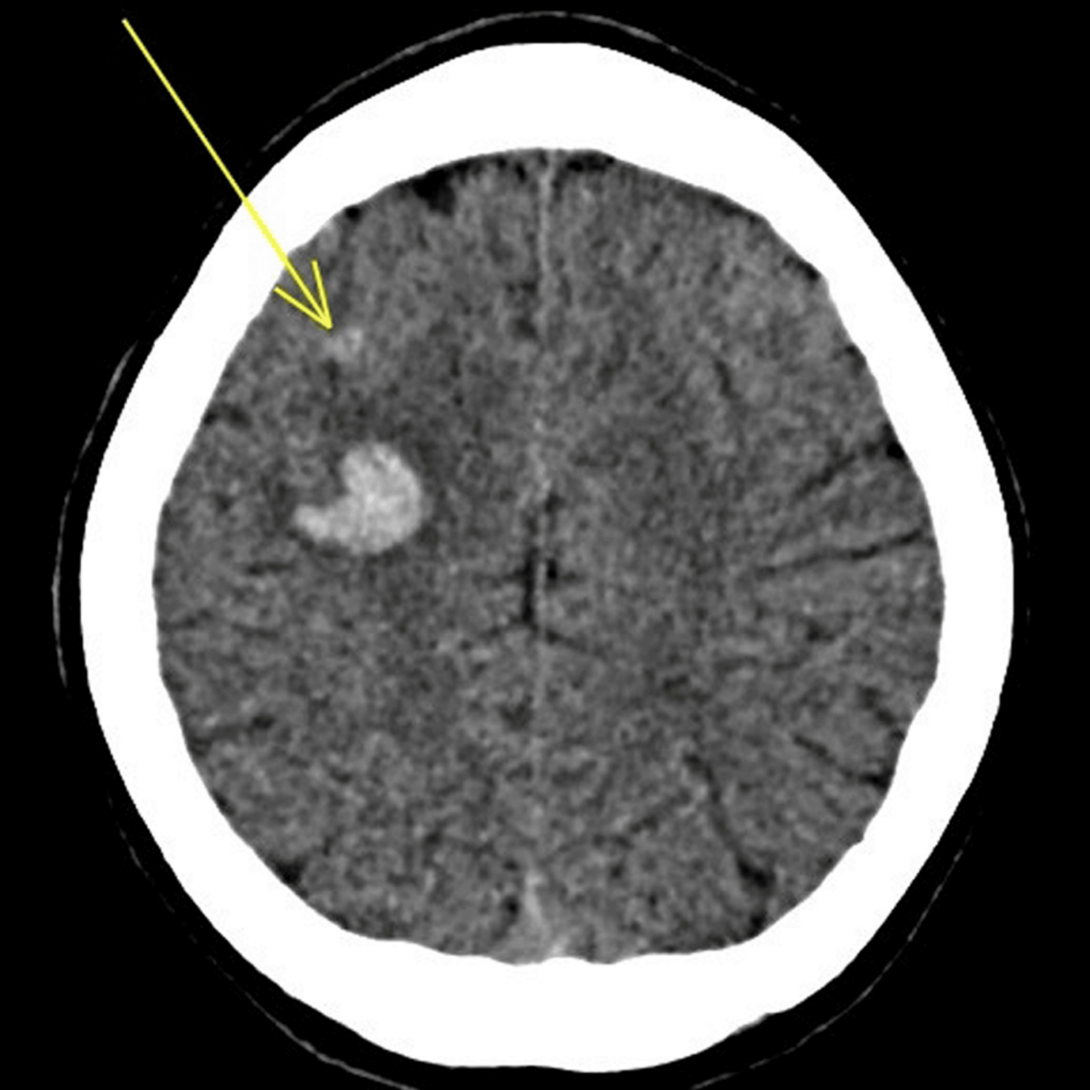
Axial CT head imaging showing a small area of brain micro-haemorrhage, highlighted by the yellow arrow

Figures 1-3 show several areas of bilateral frontal acute intraparenchymal hemorrhages with significant mass effect, the larger areas of ICH had surrounding low-volume oedema with sulcal effacement and slight rightward deviation of the falx by 2 mm, and there was no cerebellar herniation. The presence of intraparenchymal bleeding was considered significant hence early operative management is considered high risk. Radiological assessment raised concerns about mycotic aneurysms or encephalitis rather than trauma.

**Figure 4:**
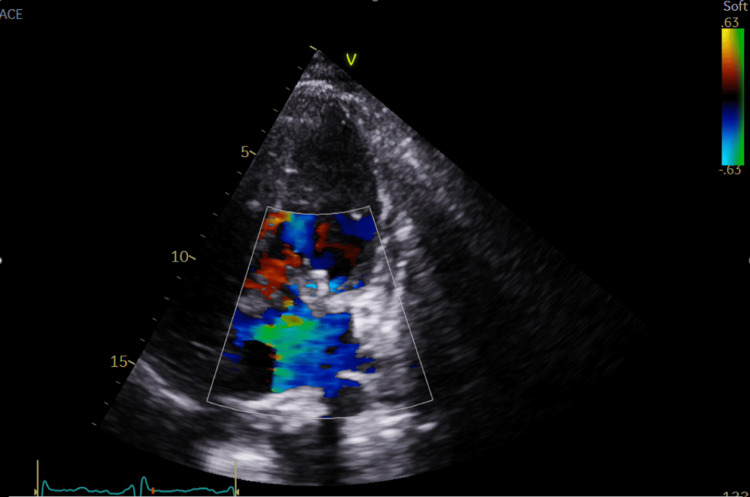
Shows significant mitral regurgitation

**Figure 5:**
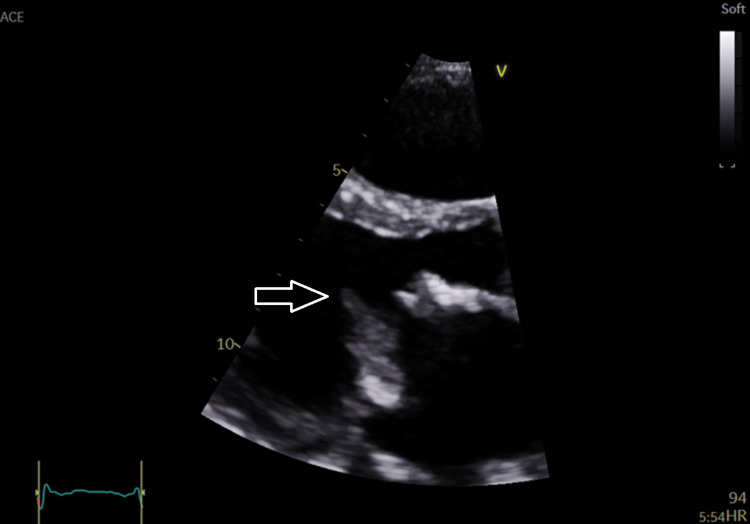
Shows PLAX view of prosthetic valve vegetations (white arrow) PLAX: parasternal long-axis

**Figure 6:**
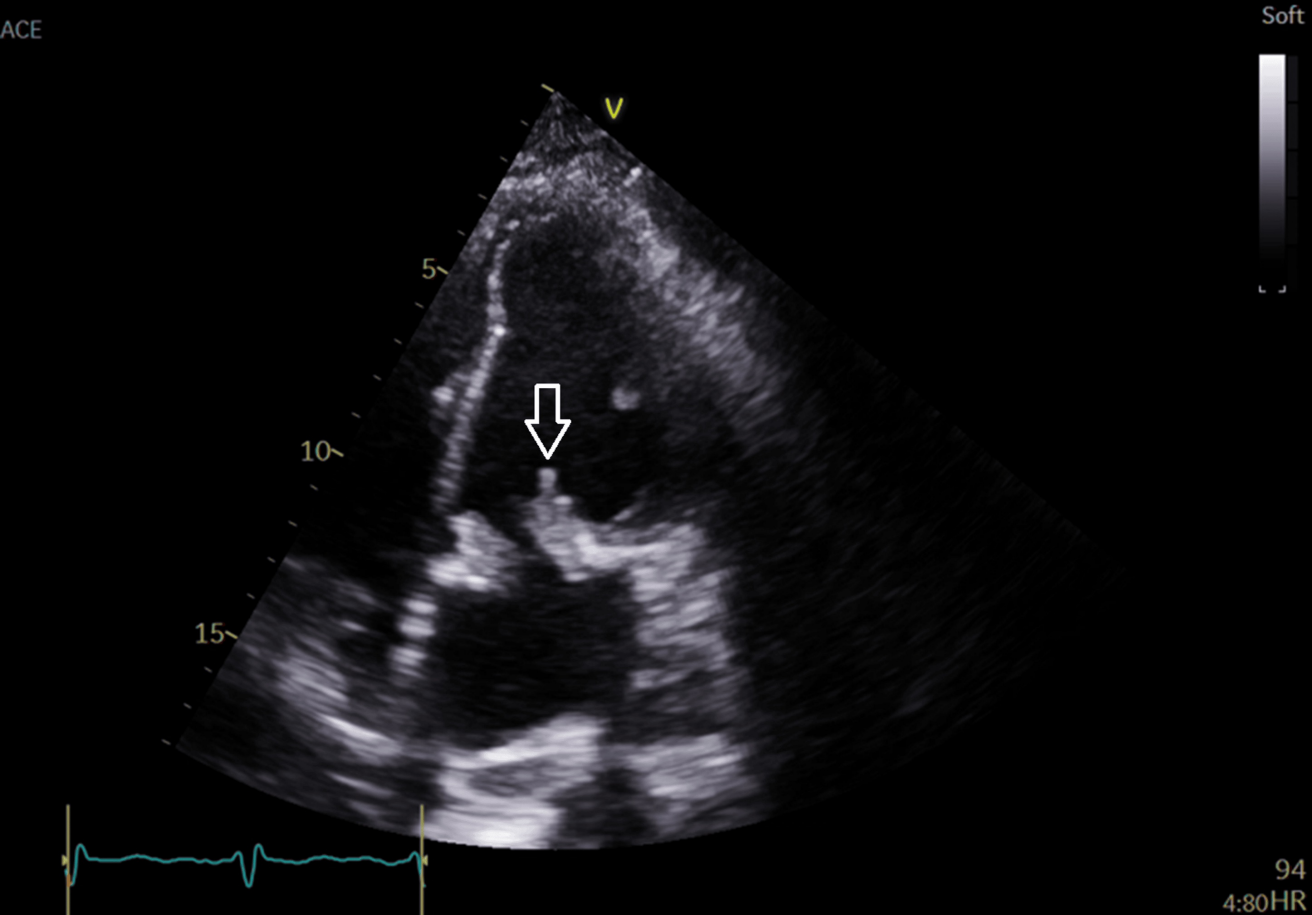
Shows AP4C view with prosthetic valve vegetations (white arrow) AP4C: apical four-chamber

**Figure 7:**
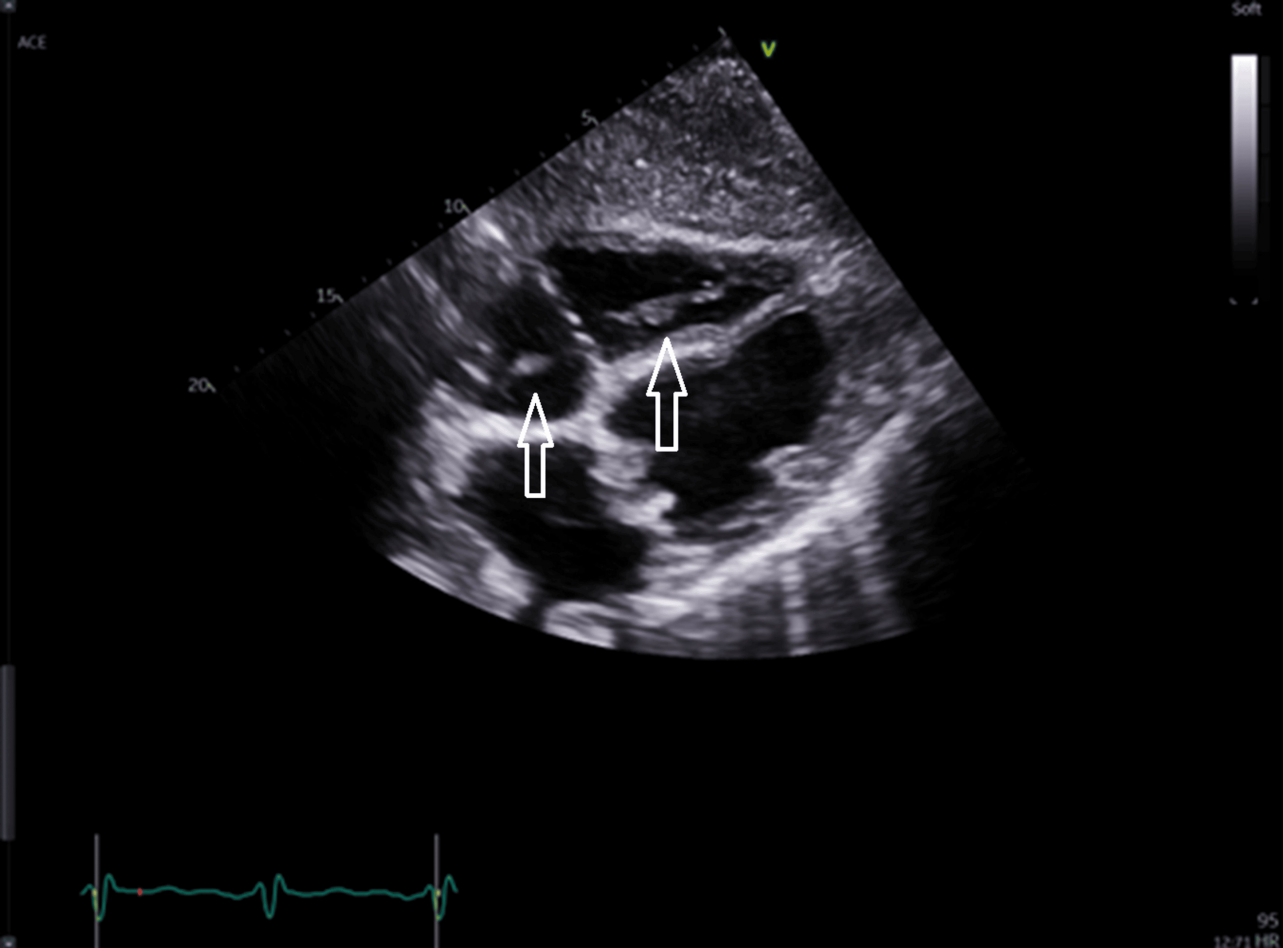
Shows cardiac device-related vegetations (white arrows)

**Figure 8:**
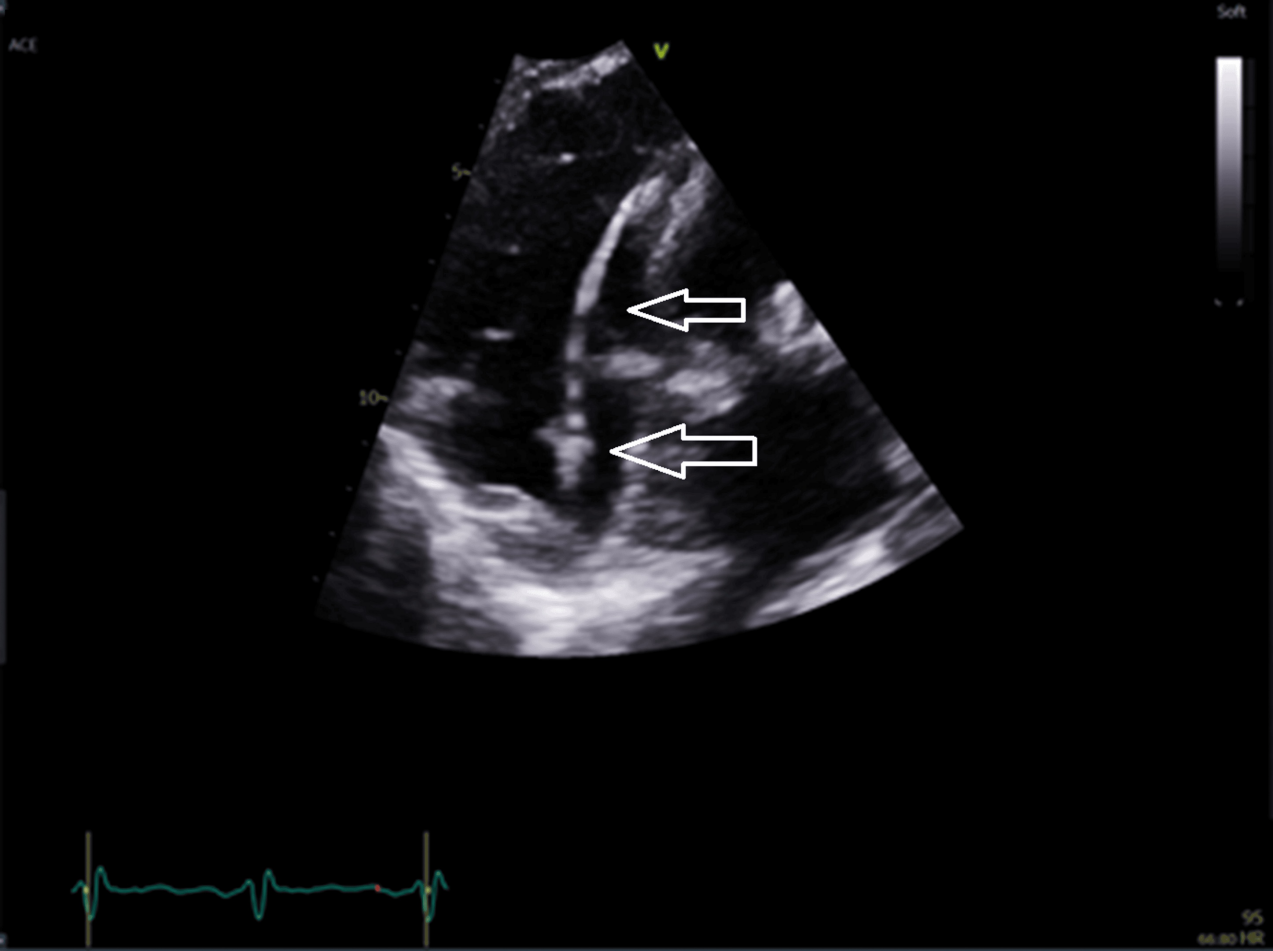
Shows cardiac device vegetations (white arrows)

The transthoracic echocardiogram (TTE) seen in Figure 4 shows mitral regurgitation. Though the degree of mitral regurgitation was difficult to quantify, it appears significant and very broad at the base. There was a high probability of pulmonary hypertension based on the peak tricuspid regurgitation velocity of 3.42 m/s. The presence of pulmonary hypertension and the left atrial dilation suggest severe mitral regurgitation. Figures 5-8 show valvular and cardiac device-related vegetations, which are the small echo bright regions seen on both pacing leads. The bioprosthetic valve is highly disorganized, with markedly thickened leaflets and mobile oscillating masses observed on both the atrial and ventricular sides, as highlighted by the arrows in Figures 5-6.

The presence of significant vegetation on both sides of the valve increases the patient's risk of having embolic phenomena in several organs such as the brain, bowels and spleen. Oscillating movement is a key feature of vegetation as they move with the flow of blood and the contraction of the heart, which is a distinctive characteristic compared to thrombus or other fixed masses, this also correlates with embolic risk. Unfortunately, MRI was not possible locally due to incompatibility with PPM and was planned at a different area.

The patient required transfer to the intensive care unit for vasopressor support due to hemodynamic instability. Neurosurgery recommended treatment akin to a stroke. Inotropic support was initiated due to significant mitral regurgitation and declining cardiac output. The regional cardiothoracic team deemed the patient unsuitable for urgent surgical intervention, given high perioperative risks from an ICH, re-do surgery and second valve surgery, additionally, poor previous post-operative compliance history shown by continuous intravenous drug abuse and failure to follow post-surgical advice following previous valve replacement and permanent pacemaker.

Ceilings of care were established following regional cardiothoracic team advice, including no cardiopulmonary resuscitation (CPR) or advanced cardiovascular support. The patient was weaned off ionotropic support, with a transient improvement in blood pressure and inflammatory markers while on appropriate combination antibiotic therapy targeting isolated *Staphylococcus aureus*. However, clinical deterioration ensued. Inflammatory markers surged, and the patient’s oxygen saturation declined despite adequate respiratory support. The patient showed signs of systemic failure, with rising oxygen requirement, increased work of breathing and hemodynamic compromise from sepsis. Following the failure of medical management and progressive clinical decline, the decision was made to initiate palliative care and symptom control. 

## Discussion

Cardiac surgery in patients with ICH presents significant challenges due to the necessity of systemic anticoagulation during cardiopulmonary bypass, which can exacerbate bleeding and lead to neurological deterioration. A study done by Musleh et al. [10] revealed that performing cardiac surgery within 30 days of ICH was not significantly associated with increased mortality; however, it was linked to a higher risk of neurological deterioration, particularly when surgery was conducted within the first seven days following ICH onset. This does support the latest guidelines from the European Society of Cardiology to delay surgery for at least one month; these guidelines suggest delaying surgery following an ICH to minimize the risk of exacerbating neurological injury [11]. This waiting period allows for stabilization and potential resolution of hemorrhagic events. 

For patients with delayed surgical intervention, follow-up cerebral imaging with CT or MRI should be conducted within one to two weeks after an ICH, or sooner, if clinical deterioration occurs [8]. Further imaging is essential to evaluate the stability of cerebral findings and reassess the optimal timing for surgery. The European Society of Cardiology (ESC) acknowledge that the appropriate timing of surgical intervention following ICH remains a topic of debate, highlighting the need for further research in this area [8].

Numerous studies have demonstrated higher mortality rates in patients with ICH who do not undergo surgical intervention [12]. For instance, a study by Salaun et al. [12], involved 60 patients with ICH and IE, they found that conservative management was associated with significantly higher mortality compared to surgical treatment. Furthermore, a study by Nitsch et al. [13] identified *Staphylococcus aureus* infection and thrombocytopenia as significant risk factors for the development of ICH, in that study, ICH was associated with increased mortality. These findings suggest that our patient may have been predisposed to an elevated risk of developing ICH due to these underlying factors as well as an increased mortality risk. 

The presence of severe mitral regurgitation further complicated the prognosis, with surgical intervention deemed too high risk due to brain haemorrhage and systemic status which is compounded by poor post-operative compliance with post-surgical advice and in the form of continuous IVDU. Despite aggressive medical management including combination antibiotic therapy, the patient’s condition deteriorated, highlighting the challenges of treating complex multi-system diseases in the context of persistent bacteremia, and several vegetative lesions without the option of surgery.

## Conclusions

This case highlights the challenges of managing critically ill patients with overlapping cardiac and neurological challenges, particularly in IE. Early diagnosis, involving clinical evaluation, microbiological testing, and echocardiography, is crucial to initiate timely antibiotic therapy and reduce embolic and haemorrhagic complications. Patients with prosthetic valves or cardiac implantable electronic devices face unique risks for stroke due to distinct mechanisms associated with these foreign materials. These devices provide surfaces where bacteria can adhere, increasing the risk of septic emboli and other systemic complications. Neurologic symptoms, such as stroke, can be the initial presentation of IE, emphasizing the need for early recognition, especially in young patients. Surgical intervention, while sometimes necessary, poses significant risks in haemorrhagic stroke cases due to potential neurological worsening with cardiopulmonary bypass, requiring a multidisciplinary approach, and shared decision-making to balance risks, benefits and timing of surgery. Bridging strategies exist such as delayed anticoagulation and step-wise haemodynamic stabilization to optimize outcomes while awaiting surgery. When medical management is futile, transitioning to palliative care ensures a dignified end-of-life process and avoids prolonged suffering.
